# Job quality and precarious employment among lesbian, gay, and bisexual workers: A national study

**DOI:** 10.1016/j.ssmph.2023.101535

**Published:** 2023-10-20

**Authors:** David J. Kinitz, Faraz Vahid Shahidi, Lori E. Ross

**Affiliations:** a Dalla Lana School of Public Health, University of Toronto, 155 College Street, Room 500, Toronto, Ontario, M5T 3M7, Canada; b Institute for Work & Health, 400 University Avenue, Suite 1800, Toronto, Ontario, M5G 1S5, Canada

**Keywords:** Job quality, LGB, Precarious employment, Quantitative, Sexual minority, Work

## Abstract

**Background:**

Employment outcomes among sexual minority (i.e., lesbian, gay, bisexual) workers are poorly understood, and previous research on this topic has focused almost exclusively on inequities in earnings, neglecting other important dimensions of job quality. We address this gap by describing and comparing the job quality of straight and sexual minority workers in Canada.

**Methods:**

Data are from the 2016 General Social Survey: Canadians at Work and Home, the only national survey providing both a measure of sexual orientation and a multidimensional view of job quality in Canada. We identified 25 unique job quality indicators (e.g., temporary employment; job insecurity; health benefits; low income; job satisfaction; job control; discrimination). Latent class cluster analysis was used to establish a typology of job quality describing standard, flexible, and precarious employment types. We used multivariable regression methods to examine the association between sexual orientation and job quality.

**Results:**

Sexual minorities reported lower job quality than their straight counterparts along many dimensions, with bisexual people reporting the lowest job quality. While inequities were generally observed among both sexual minority men and women, they sometimes differed in magnitude by gender. The prevalence of precarious employment was nearly three times higher among lesbian, gay, and bisexual workers (PR: 2.94, CI: 1.89–4.58 among all sexual minorities; PR: 3.04, CI: 1.71–5.43 among gay/lesbian workers; and PR: 2.81, CI: 1.45–5.47 among bisexual workers) compared to their straight counterparts.

**Conclusion:**

Inequities in job quality among sexual minorities persist despite comprehensive human rights protections in Canada. These inequities are pervasive, extending well beyond conventional indicators such as dollars earned and hours worked. Multi-pronged interventions are needed that move beyond simply ensuring that sexual minority workers are employed. Sexual minority workers deserve access to secure, well-paid work with benefits where they can foster connection and be free from discrimination.

## Highlights

• Sexual minority workers report poorer job quality than straight workers.
• Bisexual workers report the poorest job quality outcomes.
• Sexual minority workers are approximately three times more likely to be precariously employed compared to straight workers.

## 1 Introduction

Sexual minority people are estimated to comprise four to seven percent of the population in Canada and the US (Jones, 2022; Statistics Canada, 2022). This large minority population faces inequities in many aspects of life, sustained by discriminatory social attitudes and policies that restrict sexual minority people’s ability to participate in society to their full potential (Mendos, 2019). Employment is a key sphere of life that has been underexplored among sexual minority people and has the potential to significantly impact mental and physical health (Benach et al., 2014; Eisenberg-Guyot et al., 2020; Peckham et al., 2019; Van Aerden et al., 2016). With the deterioration of employment conditions and the increasing prevalence of precarious employment in recent decades (Oddo et al., 2021), there is need to better understand sexual minority people’s employment outcomes with the goal of informing interventions to address social, economic, and health inequities experienced by this population. Given the significant barriers that sexual minority people face within the labour market, they may be particularly vulnerable to low job quality and precarious employment (Hollibaugh & Weiss, 2015), though no studies have empirically explored this. Moreover, sexual minorities are a heterogeneous population with diverse experiences. Heterosexism and sexism, in tandem, can shape access to opportunities and resources in the labour market for sexual minorities differently (Badgett, 2014, pp. 29–52; Ragins et al., 2003).

Little is known about the job quality of sexual minority workers, due in part to a lack of information on sexual orientation in national survey data (Waite & Denier, 2019). In Canada, for instance, there is no way of identifying sexual minority workers in the flagship Labour Force Survey, severely constraining needed insights into the job quality of these workers. As a result, existing research has focused almost exclusively on the simplest of employment indicators, such as hours worked and dollars earned. Previous studies tell us that sexual minority workers often earn less, work fewer hours, and are more likely to experience workplace discrimination relative to the general working population (Allan et al., 2020; Bayrakdar & King, 2022; Dilmaghani, 2018a; Waite et al., 2019). However, there are many other important dimensions of job quality that have yet to be explored, such as job security, access to benefits, schedule predictability, job match, job control, bullying, harassment, and many others. Given the high rates of discrimination that sexual minority workers face in the labour market, it stands to reason that these workers experience inequities across a broad set of employment and working conditions, not simply hours worked and dollars earned. These broader conditions are essential to consider given their impact on health and socioeconomic wellbeing (Andrea et al., 2022; Horowitz, 2016; Peckham et al., 2019).

This study provides the most comprehensive portrait of job quality among lesbian, gay, and bisexual workers to date. Using data from the Canadian General Social Survey (GSS), we examine how the job quality of sexual minority workers compares to that of their straight counterparts. In doing so, we highlight what we can and cannot learn from the single best source of data on the job quality of sexual minority workers in Canada.

## 2 Methods

### 2.1 Data and sample

Data are drawn from the 2016 Canadian GSS. The GSS is a cross-sectional survey administered on an annual basis to a sample of approximately 25,000 persons aged 15 years and older. Its primary objective is to gather information on the social and economic well-being of the household population in Canada. Annual cycles of the GSS are assigned a special theme. The special theme of the 2016 GSS was “Canadians at Work and Home”, with a corresponding mandate to explore people’s views on work, home, leisure, and well-being (Statistics Canada, 2017). At present, the 2016 GSS provides the single best source of information on the employment and working conditions of lesbian, gay, and bisexual workers in Canada. The response rate of the 2016 GSS was 50.8%. Response rates differed by income, race, household composition, and other sociodemographic characteristics.

Our population of interest consisted of adults 18 years of age or older and employed during the week preceding the survey interview (n = 9735). We excluded respondents who were unemployed or out of the labour force (e.g., due to schooling, caregiving, early retirement, or disability). We removed respondents who were missing information on sexual orientation (n = 120) as well as those missing information on other study variables (n = 345). This resulted in a final analytic sample of 9270 respondents. (Sample sizes are randomly rounded to the nearest five in accordance with Statistics Canada requirements.)

### 2.2 Variables

Sexual orientation was measured using the following question: “Do you consider yourself to be heterosexual, homosexual, or bisexual?” Respondents had the option not to respond or to respond that they do not know. Our analytic sample consisted of 8970 heterosexual (hereafter straight) workers and 300 non-heterosexual (hereafter sexual minority) workers. The latter group was in turn comprised of 190 homosexual (hereafter gay/lesbian) workers and 110 bisexual workers. Our choice of terminology (e.g., ‘gay’ and ‘lesbian’ instead of ‘homosexual’) reflects a responsibility and desire for researchers to move away from pathologizing language included in earlier versions of the Diagnostic and Statistical Manual of Mental Disorders (Drescher, 2015). We also employ the term ‘sexual minority’ to factually speak to the reality that lesbian, gay, and bisexual people are statistically a minority population.

Job quality was measured using a diverse pool of questions capturing twenty-five dimensions of work. Ten of the dimensions describe objective aspects of the employment relationship. The remaining fifteen dimensions describe everyday experiences on the job and in the workplace. Hereafter, we refer to these two sets of variables as “employment conditions” and “working conditions”, respectively. Respondents were asked about the following employment conditions: temporary employment; part-time employment; self-employment; irregular employment; union membership; low income; pension benefits; paid sick leave; disability benefits; and health benefits. Respondents were asked about the following working conditions: job insecurity; job satisfaction; overqualification; job mismatch; carer prospects; job demands; job control; sense of belonging at work; work-life balance; discrimination; verbal abuse; sexual harassment; threats; humiliating behaviour; and physical violence. Each of these employment and working conditions was coded as a binary indicator (e.g., temporary employment versus permanent employment; high job control versus low job control). We describe how we operationalized these indicators and their associated survey items in the online supplementary appendix (Table S1).

We also collected information on sociodemographic factors that may confound the relationship between sexual orientation and job quality. We included the following covariates in our analyses: age (years), gender (man or woman), race (white or non-white), immigrant status (born in Canada or born outside Canada), region of residence (Eastern Canada, Central Canada, Western Canada, or Northern Canada), and urbanicity (urban or rural).

### 2.3 Statistical analyses

We calculated descriptive statistics to summarize the sociodemographic characteristics of the sample and describe the distribution of study variables. We used log-binomial regression models to estimate associations between sexual orientation and each of the twenty-five job quality indicators. We employed log-binomial regression instead of the more conventional logistic regression approach due to the poor interpretability of odds ratios with common outcomes (Davies et al., 1998; Deddens & Petersen, 2008). We first compared heterosexual workers to all non-heterosexual workers. We then disaggregated the latter group in order to compare heterosexual workers to homosexual and bisexual workers separately. Next, we used latent class analysis (LCA) to construct a typology of job quality capturing all twenty-five job quality indicators. Following suit with previous literature in this area, we used LCA to group workers into distinct ‘types’ of jobs, each of which is characterized by a specific configuration of employment and working conditions (Chen & Mehdi, 2019; Peckham et al., 2022; Shahidi et al., 2023; Van Aerden et al., 2014). Because self-employed workers were excluded from answering certain job quality questions (e.g., type of employment contract, union membership, access to workplace benefits), they were not included in the LCA, leading to a smaller overall sample in this part of our analysis (n = 7880). We estimated multiple LCA models enumerating a progressively larger number of latent classes. To assess the adequacy of each model, we used fit indices (i.e., Bayesian Information Criterion, the Akaike Information Criterion, and the Vuong-Lo-Mendell-Rubin likelihood ratio test) in combination with a substantive interpretation of model results (Masyn, 2013). Upon selection of the final LCA model, we used descriptive statistics to summarize key features of the job quality typology and to calculate the prevalence of each ‘type’ of job across study groups. Finally, we used multinomial regression models to estimate associations between sexual orientation and job quality. We employed multinomial regression models because of the categorical distribution of the dependent variable (i.e., with more than two latent classes, there are more than two possible outcomes).

Prior research has noted that the association between sexual orientation and socioeconomic outcomes can vary substantially according to gender (Carpenter, 2008; Denier & Waite, 2019; Dilmaghani, 2018b; Waite et al., 2019). To supplement our main findings, we conducted additional analyses stratifying the sample and examining the relationship between sexual orientation and job quality for men and women separately. Due to the small sample of non-heterosexual men (n = 175) and non-heterosexual women (n = 125), we lacked sufficient statistical power to distinguish between homosexual and bisexual respondents within these gender-stratified samples. We exercised caution when interpreting the gender-stratified results for the same reason (i.e., small sample sizes resulting in a lack of statistical power). As noted earlier, despite the limitations posed by its small sample of sexual minority respondents, the 2016 GSS remains the single best data source for assessing the job quality of sexual minority workers in Canada.

Analyses were conducted in Stata 16.0 and Mplus 8.1. Regression estimates are presented in the form of adjusted prevalence ratios (PR) and corresponding 95% confidence intervals (CI). Regression models are adjusted for age, gender, race, immigrant status, region of residence, and urbanicity. In each model, heterosexual workers were chosen as the reference group. We incorporated survey weights provided by Statistics Canada to correct for sampling and non-response bias.

## 3 Results

### 3.1 Sociodemographic characteristics

The study sample description is presented in Table 1. Of 9270 observations, 300 were sexual minorities with 110 bisexual people and 190 gay or lesbian people. When considered in the aggregate, sexual minority workers were younger and more likely to live in an urban area, but otherwise comparable to their straight counterparts. Further disaggregation by sexual orientation revealed some notable differences between gay and lesbian and bisexual workers. Whereas most gay/lesbian participants were men (70.6%), most bisexual participants were women (61.8%). Furthermore, compared to straight and gay/lesbian workers, bisexuals workers were younger, less likely to be white, and less likely to live in Eastern Canada.

**Table 1:** Sociodemographic characteristics of the sample, weighted proportions.

Sociodemographic Characteristic	Straight	Sexual Minority		
		All Sexual Minority	Gay/Lesbian	Bisexual
**Observations**	n = 8970	n = 300	n = 190	n = 110
**Age**				
18-29	16.7%	37.0%	33.6%	42.4%
30-39	23.7%	23.0%	21.3%	25.7%
40-49	24.4%	19.0%	20.0%	17.4%
50-59	25.4%	11.9%	13.4%	9.5%
60+	9.9%	9.1%	11.8%	4.9%
Gender				
Man	55.3%	58.1%	70.6%	38.2%
Woman	44.7%	41.9%	29.4%	61.8%
**Race**				
White	75.8%	72.0%	74.6%	67.9%
Non-white	24.2%	28.0%	25.4%	32.1%
**Immigrant Status**				
Born in Canada	78.0%	82.2%	84.0%	79.5%
Born outside Canada	22.0%	17.8%	16.0%	20.5%
**Region**				
Central Canada	32.3%	36.2%	33.2%	41.0%
Eastern Canada	61.3%	57.4%	61.8%	50.5%
Western/Northern Canada	6.4%	6.3%	5.0%	8.5%
**Urbanicity**				
Urban	84.7%	92.2%	92.3%	92.2%
Rural	15.3%	7.7%	7.7%	7.8%

Note: Western and Northern Canada are combined due to the small sample of respondents from Northern Canada.

### 3.2 Job quality by sexual orientation

Job quality indicators were organized into employment and working conditions and analyzed by sexual orientation group (Table 2). With respect to employment conditions, relative to straight workers, sexual minority workers were more likely to report part-time or irregular employment and less likely to report union membership. The proportion of workers in self-employment and temporary employment were similar across the two groups. Compared to straight workers, sexual minority workers were more likely to report low income and a lack of access to workplace benefits such as a pension plan, paid sick leave, disability insurance, and supplemental health care. Further disaggregation by sexual orientation again revealed some notable heterogeneity. Gay and lesbian workers were more likely to report self-employment and irregular employment than both straight and bisexual workers alike. Bisexual workers, on the other hand, were more likely to report part-time employment. Compared to other groups, they were also less likely to report self-employment and more likely to report low income. Finally, bisexual workers reported greater access to workplace benefits than gay and lesbian workers.

**Table 2:** Job quality indicators by sexual orientation, weighted proportions.

	Straight	Sexual Minority		
Job Quality Indicator		All Sexual Minority	Gay/Lesbian	Bisexual
**Observations**	n = 8970	n = 300	n = 190	n = 110
**Employment Conditions**				
Temporary employment	9.2%	8.3%	7.0%	10.3%
Part-time employment	9.9%	16.0%	10.7%	24.2%
Self-employment	14.1%	12.9%	17.1%	6.2%
Irregular employment	20.7%	29.4%	34.2%	22.0%
Union membership	33.1%	28.1%	28.4%	27.7%
Low income	35.0%	51.8%	48.9%	56.3%
Pension benefits	41.0%	33.6%	30.3%	38.2%
Paid sick leave	44.4%	36.6%	32.1%	42.9%
Disability benefits	45.0%	38.9%	35.6%	43.4%
Health benefits	49.2%	42.3%	39.9%	45.6%
**Working Conditions**				
Job insecurity	9.9%	10.3%	7.6%	14.7%
Job satisfaction	86.1%	75.2%	72.8%	78.5%
Overqualification	16.8%	26.3%	19.7%	36.6%
Job mismatch	22.2%	34.4%	28.4%	43.7%
Low career prospects	26.8%	33.3%	30.8%	36.7%
High Job demands	6.0%	8.9%	9.2%	8.6%
High Job control	67.3%	68.3%	71.9%	63.0%
Low sense of belonging	5.5%	10.7%	8.4%	14.0%
Work-life balance	68.2%	57.0%	53.4%	62.6%
Discrimination	8.4%	18.5%	15.2%	23.7%
Verbal abuse	10.7%	16.4%	17.4%	14.8%
Sexual harassment	1.5%	8.0%	–	–
Threats	2.9%	3.6%	3.0%	4.5%
Humiliating behaviour	4.8%	11.2%	8.0%	16.0%
Physical violence	1.9%	1.5%	–	–

Note: Blank cells (−) indicate results that cannot be published due to small sample sizes, in conformation with Statistics Canada requirements.

Relative to straight workers, sexual minorities reported consistently poorer working conditions. Sexual minority workers were less likely to report job satisfaction and work-life balance, and more likely to report overqualification, job mismatch, low career prospects, and a low sense of belonging at work. On the other hand, the prevalence of job insecurity and job control was similar across the two groups – at least in the aggregate. Among sexual minority workers, bisexual workers often reported the least favourable working conditions. For example, they reported a higher prevalence of job insecurity than straight and gay and lesbian workers alike (14.7% versus 7.6% and 9.9%, respectively). Similarly, they were more likely than both other groups to report overqualification, job mismatch, and a low sense of belonging at work. Conversely, bisexual workers reported greater job satisfaction than gay and lesbian workers (78.5% versus 72.8%). This difference notwithstanding, the prevalence of job satisfaction was still highest among straight workers (86.1%).

With regard to belonging, discrimination, and abuse at work (Table 2), sexual minority workers often reported worse outcomes than straight workers, with bisexual workers reporting the greatest levels of discrimination and humiliating behaviour at work. Verbal abuse, on the other hand, was most prevalent among gay and lesbian workers. Sexual minority workers, in the aggregate, were more than five times as likely to report sexual harassment at work.

In multivariable analyses adjusted for sociodemographic factors (i.e., age, gender, race, immigrant status, region of residence, and urbanicity), several associations were observed between job quality indicators and sexual orientation (Table 3). For employment conditions, part-time employment, irregular employment, and low income were higher among sexual minorities compared to straight workers. After disaggregating gay and lesbian and bisexual responses, we found that only low income was higher in both populations. Among gay and lesbian workers, temporary employment was less likely (PR: 0.72; 95% CI: 0.30–1.74) and self-employment was more likely (PR: 1.37; CI: 0.96–1.95). Bisexual workers were 2.54 times more likely to report part-time employment (95% CI: 1.54–4.18), while gay and lesbian workers were 1.67 times more likely to report irregular employment (95% CI: 1.25–2.24). Bisexuals were less likely to be self-employed (PR: 0.54; CI: 0.24–1.21).

**Table 3:** Associations between job quality indicators and sexual orientation.

**J**ob Quality Indicator	All Sexual Minority	Gay/Lesbian	Bisexual
	PR (95% CI)	PR (95% CI)	PR (95% CI)
**Employment Conditions**			
Temporary employment	0.86 (0.47–1.57)	0.72 (0.30–1.74)	1.06 (0.48–2.35)
Part-time employment	**1.89 (1.28–2.79)**	1.40 (0.80–2.46)	**2.54 (1.54–4.18)**
Self-employment	1.09 (0.79–1.51)	1.37 (0.96–1.95)	0.54 (0.24–1.21)
Irregular employment	**1.45 (1.12–1.87)**	**1.67 (1.25–2.24)**	1.10 (0.66–1.82)
Union membership	0.91 (0.71–1.17)	0.92 (0.67–1.26)	0.90 (0.67–1.26)
Low income	**1.37 (1.19–1.58)**	**1.39 (1.16–1.66)**	**1.34 (1.08–1.67)**
Pension benefits	0.83 (0.65–1.07)	0.74 (0.52–1.06)	0.96 (0.68–1.35)
Paid sick leave	0.83 (0.66–1.05)	0.73 (0.53–1.01)	0.97 (0.71–1.32)
Disability benefits	0.87 (0.70–1.08)	0.78 (0.58–1.05)	1.00 (0.73–1.37)
Health benefits	0.86 (0.70–1.04)	0.80 (0.61–1.04)	0.94 (0.71–1.26)
**Working Conditions**			
Job insecurity	0.99 (0.58–1.71)	0.72 (0.36–1.45)	1.49 (0.69–3.21)
Job satisfaction	**0.89 (0.80–0.98)**	**0.86 (0.75–0.99)**	0.92 (0.78–1.08)
Overqualification	**1.51 (1.16–1.98)**	1.13 (0.80–1.60)	**2.13 (1.49–3.03)**
Job mismatch	**1.48 (1.17–1.88)**	1.21 (0.84–1.74)	**1.91 (1.43–2.56)**
Low career prospects	**1.37 (1.08–1.74)**	1.24 (0.92–1.68)	**1.55 (1.08–2.24)**
High Job demands	**1.55 (1.00–2.41)**	1.58 (0.92–2.73)	1.49 (0.71–3.14)
High Job control	1.03 (0.92–1.15)	1.08 (0.95–1.23)	0.95 (0.78–1.15)
Low sense of belonging	**1.89 (1.03–3.50)**	1.43 (0.63–3.27)	**2.55 (1.11–5.86)**
Work-life balance	**0.86 (0.75–0.99)**	**0.81 (0.67–0.97)**	0.96 (0.79–1.17)
Discrimination	**2.03 (1.41–2.92)**	**1.90 (1.08–3.34)**	**2.21 (1.44–3.38)**
Verbal abuse	**1.46 (1.03–2.07)**	**1.63 (1.09–2.43)**	1.22 (0.64–2.33)
Sexual harassment	**3.55 (1.83–6.90)**	–	–
Threats	1.09 (0.57–2.08)	1.03 (0.43–2.43)	1.18 (0.46–3.04)
Humiliating behaviour	1.09 (0.57–2.08)	1.72 (0.94–3.12)	**2.89 (1.55–5.38)**
Physical violence	0.72 (0.24–2.15)	–	–

Note: Reference group is heterosexuals. Model estimates are adjusted for age, sex, race, immigrant status, region, and urbanicity. Bold estimates indicate statistical significance (p < 0.05). Blank cells (−) indicate results that cannot be published due to small sample sizes, in conformation with Statistics Canada requirements.

Associations between sexual orientation and access to benefits were not statistically significant. These associations were nevertheless of notable magnitude. Sexual minorities in the aggregate were less likely to report pension benefits (PR: 0.83; CI: 0.65–1.07), paid sick leave (PR: 0.83; CI: 0.66–1.05), disability benefits (PR: 0.87; CI: 0.70–1.08), and health benefits (PR: 0.86; CI: 0.70–1.04). Further disaggregation by sexual orientation revealed that these associations were driven by gay and lesbian workers, who were less likely than all other groups to report access to these benefits.

A number of significant associations between sexual orientation and working conditions were observed. In some instances, associations were driven largely by bisexual workers, where bisexuality was a stronger predictor of overqualification, job mismatch, low career prospects, and low sense of belonging. In other instances, associations were largely driven by gay and lesbian workers, where gay or lesbian identity was a stronger predictor of lower job satisfaction, lower work-life balance, and higher verbal abuse. The only working condition where both sexual minority groups were strong predictors was discrimination. Discrimination was 2.03 times higher for sexual minority workers as a whole (95% CI: 1.41–2.92), with gay and lesbian workers reporting a 1.9 times higher (95% CI: 1.08–3.34) and bisexual workers a 2.21 times higher (95% CI: 1.44–3.38) prevalence of discrimination relative to their straight counterparts. Physical violence, on the other hand, was less likely to be reported among sexual minority workers, although this association did not meet the threshold of statistical significance (PR: 0.72; 95% CI: 0.24–2.15).

### 3.3 Job quality typologies

LCA models enumerating a progressively larger number of latent groups suggested that the optimal solution lay between three and five classes. Model solutions with more than three classes returned groups that were small in size (i.e., less than 5% of the sample) and only modest improvements in fit statistics. We therefore selected the three-class solution as our job quality typology of choice.

The job quality typology is presented in Table 4, where we describe the key characteristics of the three latent groups. We labeled the three groups as follows: standard employment (n = 3795), flexible employment (n = 3175), and precarious employment (n = 910). The standard employment group approximates the so-called ‘Standard Employment Relationship’, characterized by full-time, permanent employment offering numerous benefits and union protection. Workers in this group also reported higher levels of job satisfaction, job control, and work-life balance. At the other end of the job quality spectrum, we identified a group of precariously employed workers that deviates strongly from the ‘Standard Employment Relationship’. Workers in this group were much more likely to report part-time, temporary, or irregular employment. They were also more likely to report low income, low career prospects, high job demand, and a low sense of belonging. By contrast, they reported the lowest levels of workplace benefits, union membership, work-life balance, job control, and job satisfaction. Additionally, discrimination, verbal abuse, sexual harassment, threats, humiliating behaviour, and physical violence were all most common in this group. In short, the precarious employment group reported less favourable conditions on virtually every dimension of job quality compared to those in the standard employment type. Between the standard and precarious employment groups, a third, relatively large intermediate group was identified – a flexible employment type – whose employment conditions deviate from the standard employment group (i.e., they had nonstandard employment arrangements) with lower levels of union membership and higher probabilities of temporary and part-time work. However, unlike the precarious employment group, they reported roughly comparable or, in some instances, even better working conditions than the standard employment group, including high levels of job satisfaction, high levels of job control, and safer workplaces (i.e., the lowest rates of discrimination and abuse). This flexible employment group appears to be comprised of nonstandard but seemingly content workers who are gainfully employed and satisfied with their jobs. We ground these employment groups (and our labels for them) in existing literature in the discussion section below.

**Table 4:** Characteristics of the job quality typology, weighted proportions.

Job Quality Indicator	Job Quality Typology		
	Standard Employment	Flexible Employment	Precarious Employment
**Observations**	n = 3795	n = 3175	n = 910
**Employment Conditions**			
Temporary employment	5.0%	11.2%	19.3%
Part-time employment	4.4%	12.7%	15.7%
Irregular employment	17.2%	14.7%	31.0%
Union membership	41.9%	23.2%	30.3%
Low income	24.3%	39.6%	49.4%
Pension benefits	79.4%	4.9%	10.3%
Paid sick leave	84.3%	7.5%	10.4%
Disability benefits	90.5%	2.5%	8.1%
Health benefits	95.1%	4.9%	16.9%
**Working Conditions**			
Job insecurity	8.6%	8.1%	24.2%
Job satisfaction	90.4%	94.7%	37.6%
Overqualification	14.8%	13.7%	44.6%
Job mismatch	19.4%	21.1%	42.3%
Low career prospects	23.3%	21.0%	62.9%
High Job demands	0.6%	3.0%	16.8%
High Job control	64.1%	74.6%	44.6%
Low sense of belonging	3.8%	1.2%	28.0%
Work-life balance	71.4%	72.2%	29.6%
Discrimination	7.1%	3.0%	35.8%
Verbal abuse	10.1%	4.4%	40.4%
Sexual harassment	1.0%	0.4%	9.4%
Threats	3.1%	0.6%	10.5%
Humiliating behaviour	3.4%	0.8%	29.0%
Physical violence	2.4%	0.3%	7.7%

Note: Excludes self-employed workers.

We show the distribution of sexual orientation groups across the three job quality groups in Table 5. Compared to straight workers, sexual minority workers were more likely to be in precarious employment (28.3% versus 11.3%) and less likely to be in both standard employment (38.3% versus 47.7%) and flexible employment (32.4% versus 41.0%). Bisexual and gay and lesbian workers were roughly equally distributed across the three job quality types. Multivariate associations between job quality type and sexual orientation were then analyzed, with results presented in Table 6. Sexual minority workers were nearly three times as likely than straight workers to be in precarious employment versus standard employment (PR: 2.94, 95% CI: 1.89–4.58). This relationship was comparable for both gay and lesbian and bisexual workers. By contrast, in adjusted analyses, sexual minority workers were no more or less likely to be in flexible employment versus standard employment (PR: 1.00, 95% CI: 0.68–1.47).

**Table 5:** Job quality type by sexual orientation, weighted proportions.

Job Quality Typology	Straight	Sexual Minority		
		All Sexual Minority	Gay/Lesbian	Bisexual
**Observations**	n = 7639	n = 241	n = 146	n = 95
Standard Employment	47.7%	38.3%	37.3%	39.6%
Flexible Employment	41.0%	32.4%	34.2%	30.0%
Precarious Employment	11.3%	29.3%	28.6%	30.4%

Note: Excludes self-employed workers.

**Table 6:** Associations between job quality type and sexual orientation.

Job Quality Typology	All Sexual Minority	Gay/Lesbian	Bisexual
	PR (95% CI)	PR (95% CI)	PR (95% CI)
Flexible vs. Standard Employment	1.00 (0.68–1.47)	1.12 (0.67–1.85)	0.85 (0.47–1.56)
Precarious vs. Standard Employment	**2.94 (1.89–4.58)**	**3.04 (1.71–5.43)**	**2.81 (1.45–5.47)**

Note: Excludes self-employed workers. Reference group is heterosexuals. Model estimates are adjusted for age, sex, race, immigrant status, region, and urbanicity. Bold estimates indicate statistical significance (p < 0.05).

### 3.4 Gender-specific associations

Supplementary analysis was conducted to understand gender-specific associations between job quality indicators and sexual orientation (Table 7). In some cases, sexual minority men and women alike were more likely to report less favourable working conditions (e.g,. more likely to report overqualification, discrimination, and sexual harassment). In other instances, however, we observed some noticeable gender differences. Sexual minority men were more likely to report low income, low career prospects, and verbal abuse, whereas sexual minority women were not. Similarly, sexual minority women were more likely to report part-time and irregular employment, job mismatch, and high job demands relative to sexual minority men. Sexual minority women were 3.31 times more likely to report humiliating behaviour (CI: 1.91–5.74). Sexual minority women were more likely to report job insecurity (PR: 1.67; CI: 0.82–3.41) than straight women and sexual minority men were less likely to report job insecurity than straight men (PR: 0.63; CI: 0.30–1.34) – albeit with neither association reaching the threshold of statistically significance. Looking in turn at the job quality typology, sexual minority men and women were, respectively, 3.22 times more likely (95% CI: 1.75–5.93) and 2.71 times more likely (95% CI: 1.48–4.98) to be in precarious employment versus standard employment compared to their straight counterparts (Table 8). The difference between these gender-specific estimates was statistically insignificant, with considerable overlap in their corresponding confidence intervals.

**Table 7:** Gender-specific associations between job quality indicators and sexual orientation.

Job Quality Indicator	All Sexual Minority	Gay/Bisexual Men	Lesbian/Bisexual Women
	PR (95% CI)	PR (95% CI)	PR (95% CI)
**Employment Conditions**			
Temporary employment	0.86 (0.47–1.57)	0.62 (0.23–1.68)	1.16 (0.56–2.40)
Part-time employment	**1.89 (1.28–2.79)**	1.75 (0.92–3.34)	**1.98 (1.23–3.21)**
Self-employment	1.09 (0.79–1.51)	1.20 (0.81–1.75)	0.87 (0.47–1.61)
Irregular employment	**1.45 (1.12–1.87)**	1.31 (0.92–1.85)	**1.65 (1.14–2.41)**
Union membership	0.91 (0.71–1.17)	0.73 (0.48–1.09)	1.13 (0.83–1.53)
Low income	**1.37 (1.19–1.58)**	**1.70 (1.43–2.02)**	1.02 (0.82–1.27)
Pension benefits	0.83 (0.65–1.07)	**0.67 (0.45–0.99)**	1.05 (0.77–1.43)
Paid sick leave	0.83 (0.66–1.05)	**0.61 (0.42–0.89)**	1.10 (0.86–1.42)
Disability benefits	0.87 (0.70–1.08)	0.74 (0.68–1.22)	1.05 (0.79–1.41)
Health benefits	0.86 (0.70–1.04)	0.70 (0.72–1.19)	1.07 (0.83–1.38)
**Working Conditions**			
Job insecurity	0.99 (0.58–1.71)	0.63 (0.30–1.34)	1.67 (0.82–3.41)
Job satisfaction	**0.89 (0.80–0.98)**	**0.83 (0.71–0.97)**	0.95 (0.84–1.08)
Overqualification	**1.51 (1.16–1.98)**	**1.42 (1.00–2.03)**	**1.65 (1.01–1.31)**
Job mismatch	**1.48 (1.17–1.88)**	1.22 (0.84–1.79)	**1.85 (1.39–2.47)**
Low career prospects	**1.37 (1.08–1.74)**	**1.46 (1.07–1.99)**	1.27 (0.88–1.83)
High Job demands	**1.55 (1.00–2.41)**	1.01 (0.49–2.09)	**2.20 (1.26–3.85)**
High Job control	1.03 (0.92–1.15)	1.04 (0.89–1.21)	1.02 (0.87–1.20)
Low sense of belonging	**1.89 (1.03–3.50)**	1.85 (0.84–4.05)	1.94 (0.75–4.99)
Work-life balance	**0.86 (0.75–0.99)**	0.89 (0.75–1.06)	0.83 (0.67–1.03)
Discrimination	**2.03 (1.41–2.92)**	**2.09 (1.17–3.73)**	**1.98 (1.26–3.10)**
Verbal abuse	**1.46 (1.03–2.07)**	**1.68 (1.08–2.62)**	1.23 (0.70–2.17)
Sexual harassment	**3.55 (1.83–6.90)**	**7.56 (1.95–29.22)**	**2.88 (1.37–6.07)**
Threats	1.09 (0.57–2.08)	0.89 (0.34–2.34)	1.31 (0.55–3.14)
Humiliating behaviour	1.09 (0.57–2.08)	1.16 (0.60–2.24)	**3.31 (1.91–5.74)**
Physical violence	0.72 (0.24–2.15)	0.59 (0.08–4.32)	0.80 (0.22–2.94)

Note: Reference group is heterosexuals. Model estimates are adjusted for age, sex, race, immigrant status, region, and urbanicity. Bold estimates indicate statistical significance (p < 0.05).

**Table 8:** Gender-specific associations between job quality type and sexual orientation.

Job Quality Type	All Sexual Minority	Gay/Bisexual Men	Lesbian/Bisexual Women
	PR (95% CI)	PR (95% CI)	PR (95% CI)
Flexible vs. Standard Employment	1.00 (0.68–1.47)	1.15 (0.68–1.96)	0.91 (0.53–1.54)
Precarious vs. Standard Employment	**2.94 (1.89–4.58)**	**3.22 (1.75–5.93)**	**2.71 (1.48–4.98)**

Note: Excludes self-employed workers. Reference group is heterosexuals. Model estimates are adjusted for age, sex, race, immigrant status, region, and urbanicity. Bold estimates indicate statistical significance (p < 0.05).

## 4 Discussion

Our study provides the most comprehensive overview of job quality among sexual minority workers to date and presents evidence of persistent job quality inequities among sexual minority people in Canada. The findings indicate that sexual minority workers experience lower quality employment and working conditions relative to straight workers, with some particularly pronounced inequities observed among bisexual workers. Taking an overall view of job quality, lesbian, gay, and bisexual workers were approximately three times as likely to be in precarious employment compared to straight people. Taking a typological view of job quality, our study also provides a multidimensional conceptualization of precarious employment that allowed us to identify the most vulnerable group of workers (Van Aerden et al., 2014; Vives et al., 2020). This multidimensional typology of job quality extends well beyond existing research on the employment and working conditions of sexual minority workers, which, to date, has primarily considered single-variable measures, such as income, employment status, and number of hours worked (see (Allan et al., 2020; Bayrakdar & King, 2022; Carpenter, 2008; Dilmaghani, 2018a; Dilmaghani, 2020; Waite et al., 2019). Inequities in job quality among sexual minorities persist despite comprehensive human rights protections for sexual minorities in Canada (Government of Canada, 2022).

The three employment groups that we identified stem from a rich body of literature that defines the ‘Standard Employment Relationship’ and highlights how flexible (or non-standard) and precarious employment deviate from this model employment relationship, particularly with respect to income, security, rights, and protections (Benach et al., 2014; Kreshpaj et al., 2020; Tompa et al., 2007; Vanroelen, 2019). That the three groups form a ‘continuum’ of job quality – ranging from standard to precarious, with an intermediate ‘flexible’ group – is also well reflected in previous studies that have employed similar typological methods to describe the nature and character of contemporary employment and working conditions (Chen & Mehdi, 2019; Peckham et al., 2022; Shahidi et al., 2021; Shahidi et al., 2023; Van Aerden et al., 2014).

Our multidimensional approach points to the need to adopt a broader view of labour market inequities among sexual minority workers (Andrea et al., 2022; Chen & Mehdi, 2019). Studies analyzing employment status – employed, unemployed, or not in the labour market – while important, do not provide a nuanced understanding of labour market inclusion of sexual minority people. It is important to distinguish between concepts of employment status and job quality when measuring labour market inclusion, since having a job is not the same as having a good quality job (Burgard & Lin, 2013; Shahidi et al., 2023). With this approach, we were able to identify both where job quality gaps exist and where these gaps are most pronounced. For example, there were differences in what we labeled as working conditions (generally more subjective variables, such as overqualification or negative perceptions of career prospects) and employment conditions (generally more objective variables, such as part-time employment, irregular employment, or low income) where we observed larger gaps in the former than the latter. Furthermore, the typological approach allowed us to organize complex and interacting dimensions of work to demonstrate how distinct types of work are unevenly distributed among worker groups, including sexual minority people (Chen & Mehdi, 2019; Peckham et al., 2022; Van Aerden et al., 2014). Our typological approach also revealed that inequities in overall job quality are considerably larger than inequities along any single dimension of job quality, reinforcing the importance of adopting a comprehensive and holistic view of employment and working conditions.

Understanding job quality among sexual minority workers is particularly important given that sexual minority populations experience disproportionate rates of poverty (Kia et al., 2019), barriers to accessing social welfare programming (Daley et al., 2023), and barriers to entering the labour market (Dilmaghani & Robinson, 2022), that may make good quality employment a more pressing issue. Further, specific variables explored, such as being part of a union, access to sick days and disability leave, and pension benefits may be particularly important for sexual minority populations. As our results show, sexual minorities face disproportionate rates of discrimination and harassment, but without good quality employment with union membership or paid time off, workers might not feel they are able to address these workplace violations. The high rates of depression, anxiety, and suicidality that sexual minorities experience, exacerbated by economic insecurity, further increase the need for secure employment with sick leave and disability benefits (Kinitz, Salway, et al., 2022). Lastly, sexual minority people often have fewer supports, and upon entering retirement may need to more heavily rely on pension benefits (Boulé et al., 2020; Emlet, 2016). The unique experiences of sexual minority people – inclusive but not limited to poor mental health, limited social and financial supports, and labour market discrimination - require attention in labour and occupational health research.

### 4.1 Dearth of sexual minority population data

One of the principal findings of this study is what *cannot* be understood about the job quality of sexual minority workers in the current data landscape. There are numerous aspects of job quality that we are unable to assess due to limitations in the available data (e.g., hourly wages, occupational health and safety outcomes, the voluntariness of variables like part-time employment). Furthermore, we identified differences in job quality between sexual minority men and women; however, due to small sample sizes, estimated differences were imprecise and often failed to meet the threshold of statistical significance. There is also inadequate representation of sexual minority people in general population surveys to enable an understanding of how sexual orientation interacts with other population characteristics, such as race, immigration status, and disability, to produce inequities at the intersections of multiple marginalized social positions. For such intersectional analyses, considerably larger samples of sexual minority workers are required.

This ‘small sample’ problem in research on sexual minorities is endemic to the Canadian data landscape, where flagship social and economic surveys (e.g., Census of Population, Canadian Income Survey, Labour Force Survey) have or do not collect information on sexual orientation (Appiah et al., 2021; Ross et al., 2019; Waite & Denier, 2019). This results in significant challenges to examine relevant variables to assess job quality by sexual orientation. For example, researchers in Canada and the US have often relied on census data, commonly understood as the gold standard for measuring employment and earnings. However, because these data sources lack explicit measures of sexual orientation, gay and lesbian people have historically been identified through household living arrangement, obfuscating and erasing bisexual people (Waite & Denier, 2019). Gender diverse people, including trans and nonbinary people, are also often overlooked in gold standard data collection methods; gender and sex are often conflated, rendering trans and non-binary people invisible. Ultimately, these reflections point to the need for a more explicit and intentional focus on sexual minority populations in survey development and data collection in Canada and peer jurisdictions. Such investments in the data landscape are vital for improving our understanding of labour market inequities and informing effective policies, programs, and protections to reduce them. Regular and routine data collection on sexual minority workers will also enable a view of how inequalities are evolving over time.

### 4.2 Sexual minority precarious employment

Grey literature has identified myriad reasons why sexual minorities may be more vulnerable to precarious employment, such as discrimination in education, hiring, and promotion practices, greater rates of policing and incarceration, increased involvement in child welfare systems, and high rates of homelessness and poverty (Hollibaugh & Weiss, 2015; Hunt & Moodie-Mills, 2012; Waite et al., 2019). Further, qualitative research has identified that sexual minority workers might self-select into jobs or sectors that are known to be less hostile and discriminatory towards sexual minority people but might be more precarious in nature (e.g., arts, retail, service) (Kinitz et al., 2022; Kinitz, 2023). Experimental audit studies have also found that sexual minority workers were discriminated against in hiring processes in various employment sectors (Dilmaghani & Robinson, 2022; Tilcsik, 2011).

Discrimination in the labour market has been well documented across many populations and is associated with poorer employment outcomes (Berghs & Dyson, 2022; Jones, 2008; Waite et al., 2019). Sexual minority people, specifically, are discriminated against in hiring and promotion (Bachmann & Gooch, 2018; Badgett et al., 2019; Dilmaghani & Robinson, 2022), experience high rates of harassment and discrimination at work (Waite, 2021), and face barriers to job tenure (Laurent & Mihoubi, 2017). This hostility and discrimination in the labour market shapes the employment outcomes and job quality of sexual minority workers (Kinitz et al., 2022; Kinitz, 2023).

### 4.3 Limitations

As identified in the results and discussion, the data are the primarily limitation of this study. The small sample size of sexual minority populations in surveys that collect data on job quality limit what can be said about job quality among this population. Moreover, trans and nonbinary workers are completely excluded and analyses considering intersections of gender, race, immigration status, and ability are either impossible or must be interpreted with extreme caution. The gender-stratified analyses conducted in this study were statistically underpowered due to the small sample, resulting in only cautious inferences based on this part of the analysis. The small sample also impacted our ability to examine how sexual minority workers are distributed across sub-types of self-employment, though previous research points to considerable heterogeneity in employment and working conditions among self-employed workers (Peckham et al., 2022). For a more precise picture, larger samples of sexual minority men and women are needed. Beyond gender, we were unable to conduct analyses on how sexual orientation intersects with other axes of oppression related to race, immigration status, and disability status. Until better data with larger samples is collected, we cannot substantiate the likelihood of heterogeneity that is likely masked within sexual minority working populations.

### 4.4 Implications

This study has implications for both population data collection and labour market interventions. It demonstrates the need to collect sexual orientation and gender identity data in national surveys to adequately monitor the employment outcomes of all sexual and gender minority populations to better inform policies, laws, and programs that might improve employment and working conditions of sexual minority workers. Our findings are clear: sexual minorities are more likely to be precariously employed. It is necessary to consider initiatives to address low job quality among sexual minority populations through multi-pronged interventions at the individual, employer, and social policy levels, as well as collect adequate data to monitor the efficacy of interventions over time. Our analysis of 25 distinct yet interconnected variables can be used to inform interventions to improve employment outcomes of sexual minority workers beyond attachment to the labour market. We show that interventions must extend beyond attachment to the labour market and consider a multiplicity of job quality indicators, such as job security, discrimination, earnings, job match, career prospects, and sense of belonging, among others.

Canada may be viewed as an example for similar equity seeking OECD countries given its strong employment protections and human rights laws that protect persons on the grounds of sexual orientation and gender identity and expression. Several other OECD countries, such as the United Kingdom (UK) and Australia, have comparable protections in place, though, similar to Canada, have been delayed in protections for trans and gender diverse people. Noteworthy, even in Canada, and with various layers of protections in place, we continue to see disparities in job quality that are captured in this study. More insights into why these inequities persist and how they can be meaningfully redressed is needed.

## Author contributions

All authors contributed to the study conception and design. Data analysis was led and executed by FVS. FVS led data interpretation with contributions from DJK and LER. The first draft of the manuscript was written by DJK with methods written by FVS. All authors commented on and made substantive contributions to the manuscript. All authors read and approved the final manuscript.

## Funding

This article draws on research supported by the Social Sciences and Humanities Research Council (grant 892-2020-2046).

## Ethics approval

All procedures performed in studies involving human participants were in accordance with the ethical standards of the institutional and/or national research committee and with the 1964 Helsinki Declaration and its later amendments or comparable ethical standards. Ethics approval was not sought for this secondary analysis.

## Declaration of competing interest

The authors declare that they have no known competing financial interests or personal relationships that could have appeared to influence the work reported in this paper.

## Data Availability

Publicly available data were used through the Research Data Centre at the University of Toronto.
