# Supplementary material for: Job quality and precarious employment among lesbian, gay, and bisexual workers: A national study
Source: SSM Popul Health. 2023 Oct 20;24:101535. doi: 10.1016/j.ssmph.2023.101535 (PMC10661442; doi:10.1016/j.ssmph.2023.101535)
Supplement: Multimedia component 1 [file mmc1.docx]

| **Table S1: Operationalization of job quality indicators from the General Social Survey (2016)** | | |
| --- | --- | --- |
| **Job Quality Indicator** | **Prompt** | **Operationalization** |
| Temporary employment | “Which of the following best describes your terms of employment in this job?” | 0: "Regular employee" 1: "Seasonal employee", "Term employee", "On-call or casual employee" |
| Part-time employment | “How many hours a week do you usually work at this job?” | 0: 30 or more hours per week 1: Less than 30 hours per week |
| Self-employment | “Were you mainly a paid worker, self-employed or an unpaid family worker?” | 0: "Paid worker" 1: "Self-employed" |
| Irregular employment | “Which of the following best describes your usual work schedule at your job?” | 0: "Regular day shift", "Regular evening shift", "Regular night shift" 1: "Rotating shift", "Split shift", "Compressed work week", "On call or casual", "Irregular schedule" |
| Union membership | “Are you a union member or covered by a union contract or collective agreement in this job?” | 0: "No" 1: "Yes" |
| Low income | Personal income was measured using official tax records. | 0: Two thirds of median personal income or more in the population 1: Less than two thirds of median personal income in the population |
| Pension benefits | "Which of the following employment benefits do you have access to a part of your employment? Pension benefits." | 0: No 1: Yes |
| Paid sick leave | "Which of the following employment benefits do you have access to a part of your employment? Paid sick leave." | 0: No 1: Yes |
| Disability benefits | "Which of the following employment benefits do you have access to a part of your employment? Disability insurance." | 0: No 1: Yes |
| Health benefits | "Which of the following employment benefits do you have access to a part of your employment? Supplemental medical or dental care." | 0: No 1: Yes |

| **Job Quality Indicator** | **Prompt** | **Operationalization** |
| --- | --- | --- |
| Job insecurity | “To what extent do you agree or disagree with the following statement? You might lose your job in the next 6 months.” | 0: "Strongly disagree", "Disagree", "Neither agree nor disagree" 1: "Agree", "Strongly agree" |
| Job satisfaction | “In general, how satisfied are you with your job?” | 0: "Very dissatisfied", "Dissatisfied", "Neither satisfied nor dissatisfied" 1: "Satisfied", "Very satisfied" |
| Overqualification | “Do you feel over-qualified, adequately qualified or under-qualified for your job?” | 0: "Adequately qualified", "Under-qualified" 1: "Over-qualified" |
| Job mismatch | “Which of the following best describes your skills in your current job?” | 0: "[My] skills are a good match for [my] job" 1: "[I] need further training to cope well with [my] duties", "[I] have the skills to cope with more demanding duties" |
| Low career prospects | “To what extent do you agree or disagree with the following statement? Your job offers good prospects for career advancement.” | 0: "Strongly agree", "Agree", "Neither agree nor disagree" 1: "Disagree", "Strongly disagree" |
| High Job demands | “How often do you consider your workload manageable?” | 0: "Always", "Often", "Sometimes" 1: "Rarely", "Never" |
| High Job control | “How often can you choose the sequence of your tasks?” | 0: "Never", "Rarely", "Sometimes" 1: "Often", "Always" |
| Low sense of belonging | “To what extent do you agree or disagree with the following statements? I feel like I belong in the organization I work for.” | 0: "Strongly agree", "Agree", "Neither agree nor disagree" 1: "Disagree", "Strongly disagree" |
| Work-life balance | “How satisfied are you with the balance between your job and home life?” | 0: "Very dissatisfied", "Dissatisfied", "Neither satisfied nor dissatisfied" 1: "Satisfied", "Very satisfied" |
| Discrimination | "In the past 12 months, have you experienced unfair treatment or discrimination while at work?" | 0: "No" 1: "Yes" |

| **Job Quality Indicator** | **Prompt** | **Operationalization** |
| --- | --- | --- |
| Verbal abuse | "In the past 12 months, have you been subjected to verbal abuse while at work? " | 0: "No" 1: "Yes" |
| Sexual harassment | "In the past 12 months, have you been subjected to unwanted sexual attention or sexual harassment while at work?" | 0: "No" 1: "Yes" |
| Threats | "In the past 12 months, have you experienced threats to your person while at work?" | 0: "No" 1: "Yes" |
| Humiliating behaviour | "In the past 12 months, have you experienced humiliating behaviour while at work?" | 0: "No" 1: "Yes" |
| Physical violence | "In the past 12 months, have you experienced physical violence while at work?" | 0: "No" 1: "Yes" |
